# Retraction: ‘Long non-coding RNA LINC00346 contributes to cisplatin resistance in nasopharyngeal carcinoma by repressing miR-342-5p’ (2020), by Cui *et al.*

**DOI:** 10.1098/rsob.230400

**Published:** 2023-11-03

**Authors:** Open Biology editorial team

**Keywords:** LINC00346, miR-342-5p, nasopharyngeal carcinoma, cisplatin resistance, lncRNA

*Open Biol.* **10**, 190286 (Published online 13 May 2020). (https://dx.doi.org/10.1098/rsob.190286)

Following an investigation, we have found that the published manuscript has substantial overlap with published manuscript [1] in the text and figures (notably fig. 6 ‘MiR-342-5p over-expression impairs resistance to cisplatin in NPC cells. (*e*) HNE1/DDP and CNE2/DDP cells transfected with miR-342-5p mimics or miR-NC were treated with various doses of cisplatin for 48 h to determine the IC50’.). This paper has been identified among of a set of papers that present significant text and data duplication and therefore the data reported in this article are unreliable. The authors have not responded to the retraction.

Prof. Jon Pines FRS

Editor-in-Chief, *Open Biology*.
